# Short and Long-Term Outcomes of Robotic versus Laparoscopic Total Mesorectal Excision for Rectal Cancer: A Case-Matched Retrospective Study

**DOI:** 10.1097/MD.0000000000000522

**Published:** 2015-03-20

**Authors:** Min Soo Cho, Se Jin Baek, Hyuk Hur, Byung Soh Min, Seung Hyuk Baik, Kang Young Lee, Nam Kyu Kim

**Affiliations:** From the Department of Surgery, Division of Colon and Rectal surgery, Yonsei University College of Medicine, Seoul, Korea.

## Abstract

The true benefits of robotic surgery are controversial, and whether robotic total mesorectal excision (R-TME) can be justified as a standard treatment for rectal cancer patients needs to be clarified. This case-matched study aimed to compare the postoperative complications and short- and long-term outcomes of R-TME and laparoscopic TME (L-TME) for rectal cancer.

Among 1029 patients, we identified 278 rectal cancer patients who underwent R-TME. Propensity score matching was used to match this group with 278 patients who underwent L-TME.

The mean follow-up period was similar between both groups (L-TME vs R-TME: 52.5 ± 17.1 vs 51.0 ± 13.1 months, *P* *=* 0.253), as were patient characteristics. The operation time was significantly longer in the R-TME group than in the L-TME group (361.6 ± 91.9  vs 272.4 ± 83.8 min; *P* < 0.001), whereas the conversion rate, length of hospital stay, and recovery of pain and bowel motility were similar between both groups. The rates of circumferential resection margin involvement and early complications were similar between both groups (L-TME vs R-TME: 4.7% vs 5.0%, *P* = 1.000; and 23.7% vs 25.9%, *P* *=* 0.624, respectively), as were the 5-year overall survival, disease-free survival, and local recurrence rates (93.1% vs 92.2%, *P* = 0.422; 79.6% vs 81.8%, *P *= 0.538; 3.9% vs 5.9%, *P* = 0.313, respectively).

The oncologic quality, short- and long-term outcomes, and postoperative morbidity in the R-TME group were comparable with those in the L-TME group.

## INTRODUCTION

With the recent progression of minimally invasive techniques for colorectal cancer, the trend of the current standard treatment of rectal cancer is going toward minimally invasive surgery (MIS). Evolution of the surgical techniques and the introduction of more advanced instruments for colorectal surgery have resulted in several advantages such as better cosmesis, quicker recovery, less postoperative pain, and a decreased hospital stay.^1–5^ Several recent randomized clinical trials have moreover demonstrated that laparoscopic total mesorectal excision (TME) shows superiority in terms of the short-term outcomes compared with open TME.^6,7^ Furthermore, a number of large-scale clinical trials have demonstrated similar outcomes between open surgery and laparoscopic surgery in the treatment of colorectal cancer in terms of their oncologic adequacy and long-term oncologic outcomes.^8–11^

Robotic systems combined with this trend have been developed as one of the treatment options for rectal cancer patients; however, to date, TME is still regarded as a technically demanding and oncologically critical procedure, especially in patients with challenging circumstances such as a narrow pelvis, lower rectal tumor, and anatomical complexity.^2,12^ Robotic surgery for rectal cancer theoretically has several advantages compared with laparoscopic surgery.^13,14^ Recently, our institution demonstrated that robot-assisted tumor-specific mesorectal excision for rectal cancer was technically feasible and a safe surgical option in terms of the long-term oncologic outcomes.^15,16^ However, there are still limited reports regarding the true benefits of robotic TME (R-TME). Furthermore, there is currently no case-matched study comparing the long-term oncologic outcomes between R-TME and laparoscopic TME (L-TME). Therefore, to clarify the true benefits of robotic surgery in the treatment of rectal cancer, this case-matched study aimed to evaluate the short- and long-term outcomes between totally robotic and L-TME for rectal cancer.

## METHODS

### Patient Selection

**FIGURE 1:**
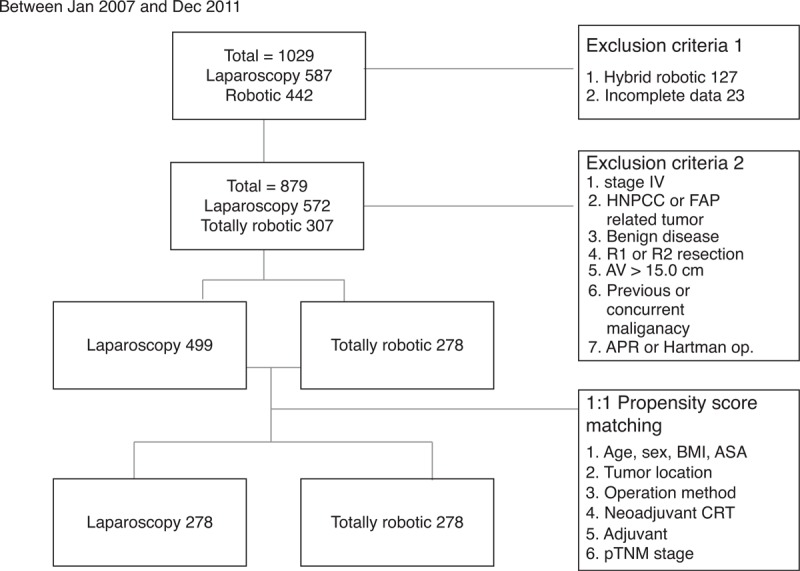
Flowchart of patient selection.

This study is a case-matched retrospective study. Between January 2007 and June 2011, a total of 1029 patients who underwent laparoscopic or robotic surgery for the treatment of colorectal disease were identified from the Yonsei Colorectal Cancer Electronic Database that covers institutional data. Among these 1029 patients, 127 patients who underwent hybrid R-TME and 23 patients with incomplete data were initially excluded. Other exclusion criteria included: stage IV disease, familial adenomatous polyposis or hereditary non-polyposis colorectal cancer, benign disease, R1 or R2 resection, tumor located >15.0 cm from the anal verge, previous or concurrent malignant disease, abdominoperineal resection, and Hartman operation. Finally, we identified 278 patients who underwent totally R-TME for rectal adenocarcinomas. Propensity score matching was used to match this group in a 1:1 ratio with 278 patients who underwent conventional L-TME. Covariates used in the logistic regression model for calculating the propensity score were age, sex, body mass index (BMI), tumor location, operation method (low anterior resection or coloanal anastomosis), neoadjuvant treatment, adjuvant treatment, and pathologic TNM stage (Figure 1). All patients were assessed preoperatively by using rectal magnetic resonance imaging (MRI), transrectal ultrasonography (TRUS), colonoscopy, and chest and abdominal computed tomography (CT). This study was approved by the Institutional Review Board of Severance Hospital.

### Surgeon Validation

All surgeries were performed by 5 surgeons of the division of colorectal surgery at Severance Hospital, Yonsei University Health System. All surgeons who performed R-TME had completed the MIS training program at the same institution. Most of the robotic cases (97.1%) were performed by 3 surgeons. Among them, 2 surgeons were primarily trained in laparoscopic colorectal surgery. One surgeon underwent laparoscopic and robotic colorectal surgery training around the same time. To assess the surgeons’ proficiency, the cumulative sum (CUSUM) method was used to quantitatively evaluate the learning curve.^17^

### Surgical Method

All patients underwent surgical treatment by either L-TME or R-TME. At our institution, we used the robotic da Vinci Surgical System (Intuitive Surgical, Sunnyvale, CA, USA) to perform R-TME via 2 different techniques: a hybrid robotic technique or totally robotic technique. In the present study, only patients who underwent totally robotic TME were included. Totally R-TME for rectal cancer surgery consists of 2 phases. The first phase is the colonic phase, which comprises inferior mesenteric vessel ligation and left colon and splenic flexure mobilization. The second phase is the pelvic phase, which constitutes pelvic dissection using the TME principles. The detailed procedures for both L-TME and R-TME have been previously described.^18,19^

### Neoadjuvant and Adjuvant Treatment

Neoadjuvant chemotherapy was performed on patients who had a tumor on the rectal wall or those with invasion to the adjacent pelvic organs. These patients with locally advanced tumors were diagnosed with adenocarcinoma via endoscopic biopsy (stage T3 or T4) and had clinically enlarged regional lymph nodes identified by TRUS and rectal MRI. For neoadjuvant chemoradiation therapy, we used a standard long-course regimen of 5-fluorouracil-based chemotherapy and a total dose of 50.4 Gy of external beam radiation. Adjuvant chemotherapy was administered based on the pathology report and by using the National Comprehensive Cancer Network guidelines.

### Patient Follow-Up

All patients were followed-up at 3-month intervals for the first 2 years. The follow-up intervals were decreased to every 6 months during the third through the fifth year, and annually thereafter. At each follow-up, the patients underwent physical examinations, blood tests with measurement of serum carcinoembryonic antigen levels, and chest radiography. Abdominopelvic CT and whole-body bone scans were performed annually. TRUS, colonoscopy, chest CT, pelvic MRI, and positron emission tomography with fludeoxyglucose were performed at the physician's discretion.

### Surgical Complications

Complications were defined as any deviations from the general postoperative course. Early and late complications were defined as postoperative complications that occurred within and after 30 days, respectively. We used a modified classification system that included 5 grades of severity to stratify surgical complications.^20^

### Recurrence Classification

The medical records of all patients were reviewed to gather information about tumor recurrence. Recurrence patterns were classified into 2 groups: local and systemic recurrence. Local recurrence was defined as any clinical or histological evidence of tumor re-growth near the primary site after the initial operation, and absence of distant metastasis. Systemic recurrence was defined as local recurrence with any distant metastasis confirmed by imaging studies or histological biopsy.

### Statistical Analysis

Propensity matching was conducted using R project for Statistical Computing, Version 2.12.0 (R Development Core Team, Vienna, Austria) along with the SPSS R Essentials plug-in. Logistic regression was used to estimate the propensity scores for each group. Eight covariates were included in the logistic regression model for calculating the propensity score. A simple nearest neighbor matching algorithm was conducted to achieve the best covariate balance after matching. Units outside the area of common support were disregarded to further improve the balance of the covariates. A Jitter plot was used for assessing the overall propensity score distribution between the groups.

Categorical variables were analyzed by using the *χ*^2^ test, and continuous variables were analyzed by using Student *t* test. The Kaplan–Meier method was used to calculate the 5-year local recurrence rate (LRR) and systemic recurrence rate (SRR), as well as the overall survival (OS), disease-specific survival (DSS), and disease-free survival (DFS) rates after surgery. OS was defined as the time from surgery to death from any cause. DSS was defined as the time from surgery to death related to cancer. DFS was defined as the time from surgery to any recurrence. All postoperative complications were analyzed using binary logistic regression. SPSS software version 20.0 for Windows (SPSS Inc, Chicago, IL, USA) was used for all analyses. All *P* values were 2-sided, and *P* < 0.05 was considered statistically significant.

## RESULTS

**FIGURE 2:**
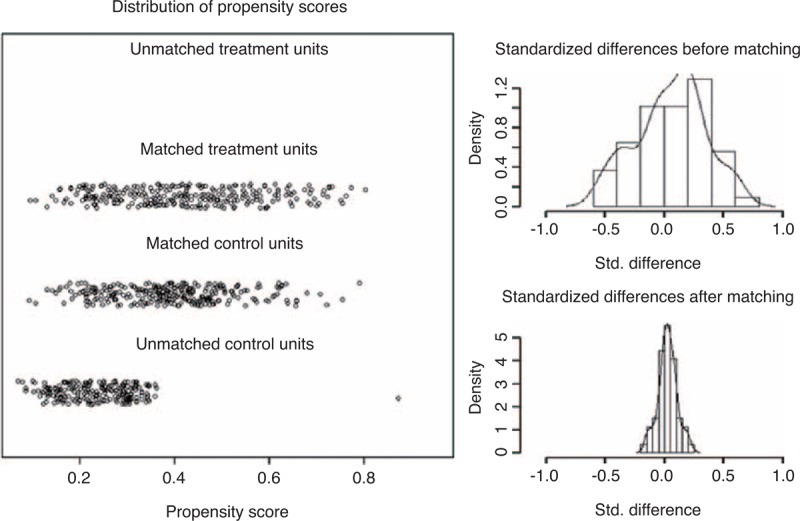
A Jitter plot of propensity score and histogram of standardized differences used for assessing the overall propensity score distribution between treatment (R-TME) and control (L-TME) groups. L-TME = laparoscopic total mesorectal excision, R-TME = robotic total mesorectal excision.

A total of 556 patients (278 matched pairs) were included in this study. The mean follow-up period was 51.8 ± 15.3 months (L-TME vs R-TME: 52.5 ± 17.1 vs 51.0 ± 13.1 months, *P* *=* 0.253). Figure 2 shows the distribution of the propensity scores of the matched treatment (R-TME) and control (L-TME) groups.

### Patient Characteristics

The demographic characteristics of the patients are presented in Table 1. There were no significant differences between the L-TME and R-TME groups in the adjusted analysis using one-to-one propensity score matching, and no relevant differences were found between the 2 groups in terms of age, sex, BMI, American Society of Anesthesiologists score, tumor location, operation method, pathologic TNM stage, history of previous abdominal surgery, mean preoperative carcinoembryonic antigen levels, and neoadjuvant or adjuvant treatment.

**TABLE 1:**
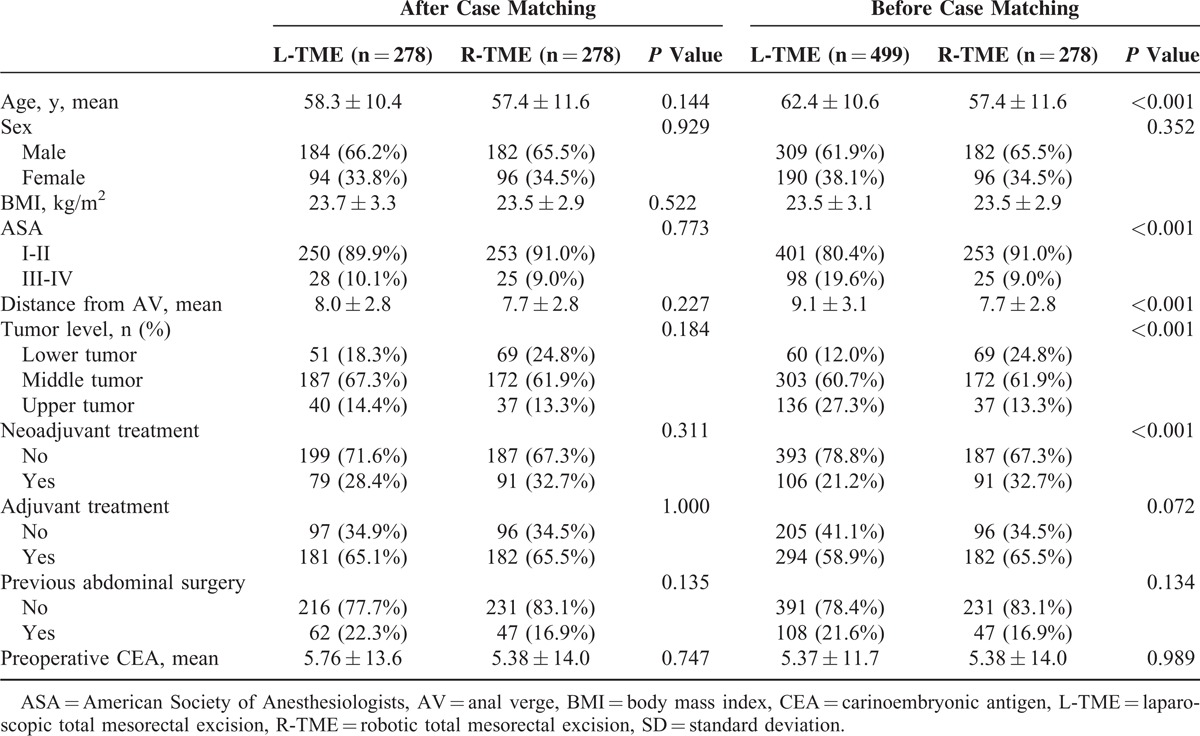
Demographic Characteristics by Using Propensity Score Matching

### Comparison of Postoperative Surgical Details and Short-term Outcomes

Comparisons of the postoperative surgical details and recovery in patients who underwent L-TME versus R-TME are summarized in Table 2. No relevant differences were found between the groups in terms of the operation method, rate of combined resection, conversion rate, and estimated blood loss. The most common resection type in both groups was low anterior resection (L-TME vs R-TME: 84.5% vs 80.9%), and the ratio of the resection type did not significantly differ between the 2 groups (*P* = 0.313). Two (0.7%) patients in the L-TME group initially had to undergo open surgery because of severe tumor adhesion and intractable major vessel bleeding. In the R-TME group, 1 (0.4%) patient was required to undergo laparoscopic surgery because of bowel perforation. The total operation time was significantly longer in the R-TME group (361.6 ± 91.9 vs 272.4 ± 83.8 min; *P* < 0.001). Combined resection in the L-TME group was performed for 14 (5.0%) patients, of whom 7, 2, 1, 1, and 1 patients underwent cholecystectomy due to gallstones, incidental appendectomy, hysterectomy due to uterine myoma, partial cystectomy due to bladder wall invasion, and extensive en bloc resections due to invasion into the small bowel wall, respectively. The remaining 2 patients underwent benign colonic mass excisions and biopsy of liver nodules. Twelve (4.3%) patients in the R-TME group underwent combined resection. Four patients underwent hysterectomy with bilateral oophorectomy owing to tumor invasion into the uterus. The other 8 patients underwent benign mass excision, including oophorectomy (n = 2), cholecystectomy (n = 3), inguinal hernioplasty (n = 1), wedge resection of the stomach due to gastrointestinal stromal tumor (n = 1), and excision of a urachal cyst (n = 1).

**TABLE 2:**
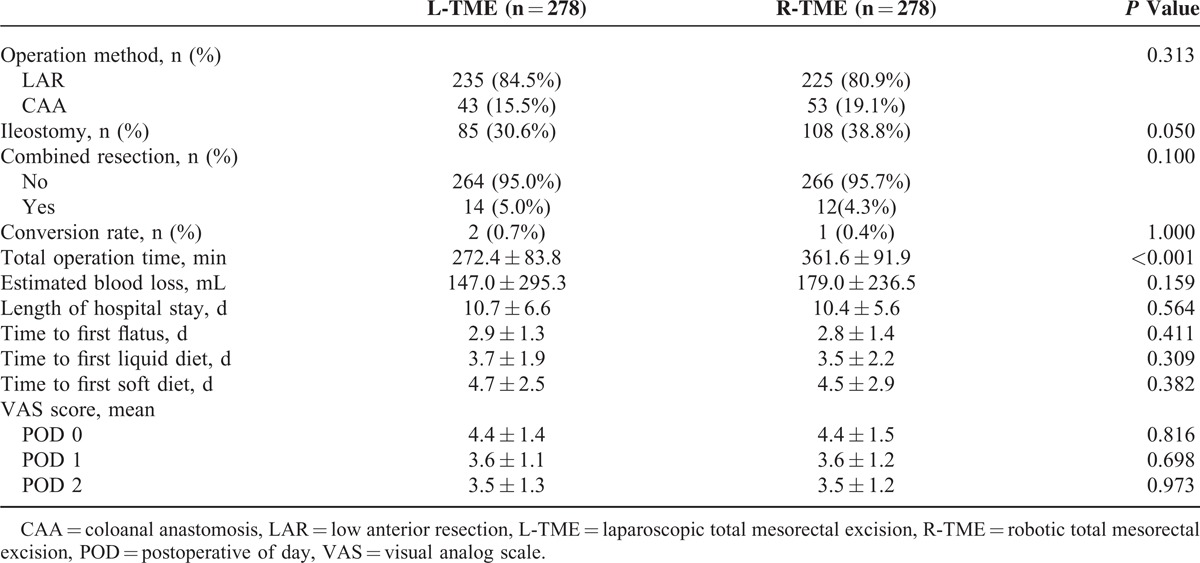
Postoperative Surgical Details and Short-term Outcomes

### Postoperative Pathologic Outcomes

The pathologic stages did not significantly differ between the groups. The mean number of retrieved lymph nodes (L-TME vs R-TME: 16.2 ± 8.1 nodes vs 15.0 ± 8.1 nodes, *P* = 0.069) and the proportion of patients with <12 nodes harvested (30.2% vs 37.8%, *P* *=* 0.073) were not significantly different between the groups. Moreover, there was no difference in the proportion of patients with a positive circumferential resection margin (CRM) (L-TME vs R-TME: 4.7% vs 5.0%; *P* = 1.000). In addition, both the mean length of the proximal and distal resection margin was not significantly different between the groups. Other pathologic outcomes, including tumor size, rate of lymphovascular invasion, grade of tumor differentiation, and pathologic T and N stage, were not significantly different between both groups (Table 3).

**TABLE 3:**
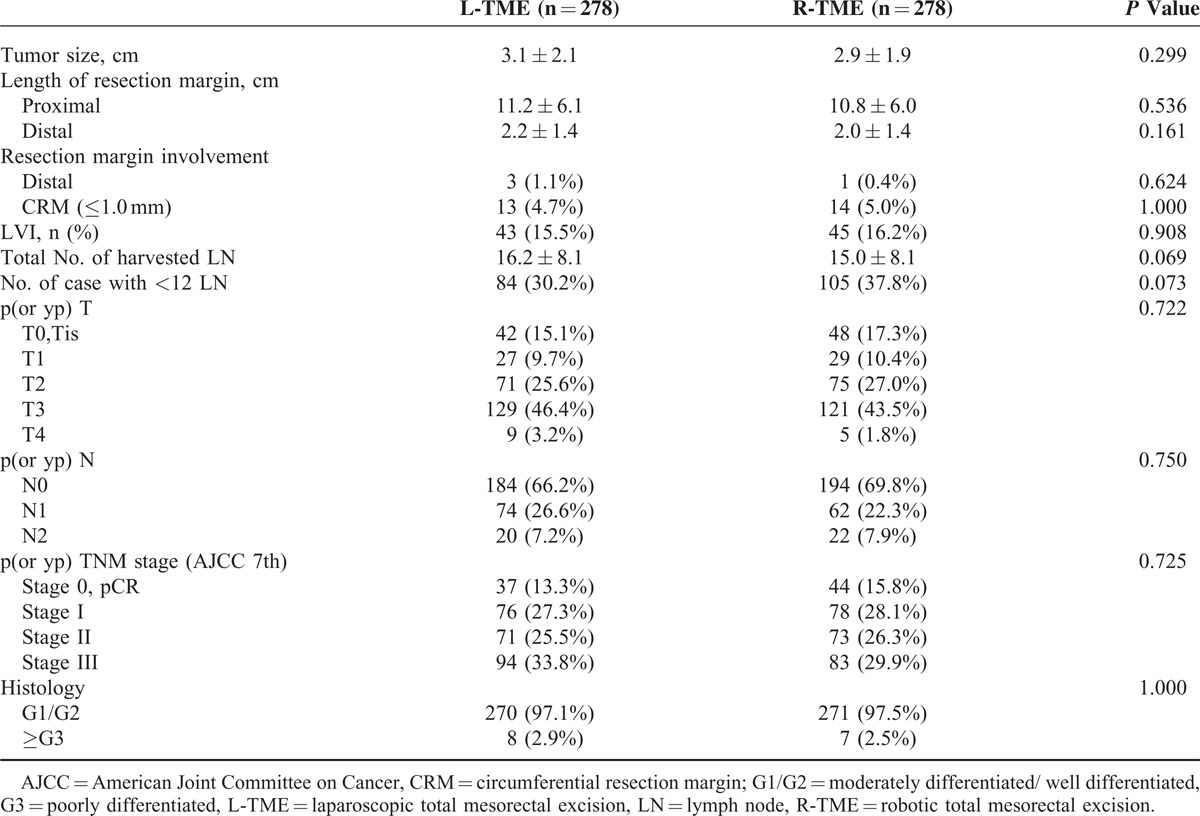
Postoperative Pathologic Outcomes

### Postoperative Complications

Both the overall early and late complications showed no significant differences between the groups. Early postoperative complications (within 30 days) occurred in 66 of 278 patients (23.7%) in the L-TME group and in 72 of 278 (25.9%) patients in the R-TME group. Anastomotic leakage was the most common type of early complication in both groups, and the rate of anastomotic leakage did not differ between the groups (L-TME vs R-TME: 10.8% vs 10.4%, *P =* 1.000). The second most common early complication in both groups was intestinal obstruction (L-TME vs R-TME: 3.2% vs 5.8%). Late complications (>30 days) occurred in 56 of 278 patients (20.1%) in the L-TME group and in 66 of 278 (23.7%) patients in the R-TME group (Table 4). The most common late complication in both groups was intestinal obstruction (L-TME vs R-TME: 5.0% vs 5.4%). In terms of functional outcomes, the rate of sexual dysfunction did not differ between the groups (L-TME vs R-TME: 2.2% vs 2.5%). However, the rate of voiding dysfunction was significantly higher in the L-TME group (4.3% vs 0.7%). According to the Clavien-Dindo classification, 32 patients (11.5%) in the L-TME group were found to have Grade I–II complications, whereas 38 patients (13.7%) in the R-TME group had Grade I–II complications. However, 34 patients (12.2%) in the L-TME group and 34 patients (12.2%) in the R-TME group were reported to have Grade III–IV complications. One patient (0.4%) in the L-TME group died of cardiac arrest during the immediate postoperative period (Table 4).

**TABLE 4:**
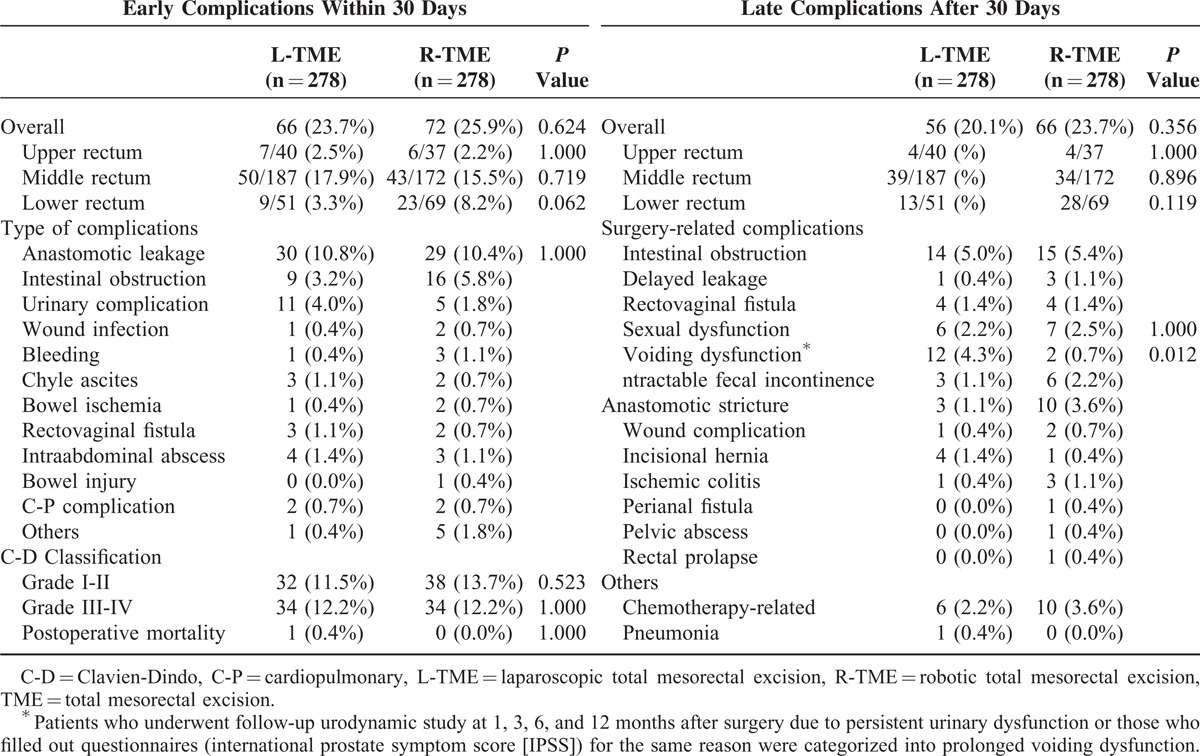
Postoperative Early and Late Complications

### Comparison of Tumor Recurrence Pattern and Long-term Oncologic Outcomes

**FIGURE 3:**
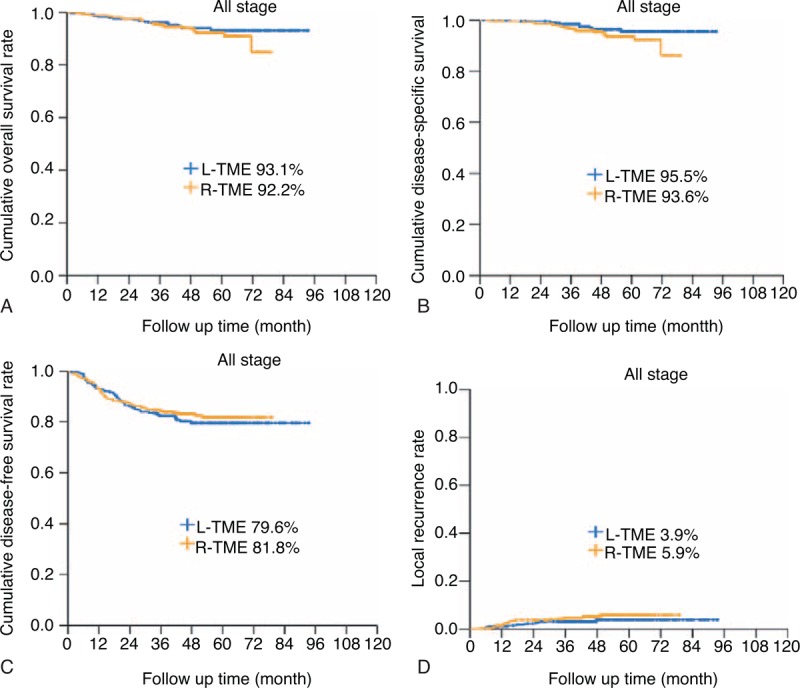
(A) 5-year overall survival, (B) disease-specific, (C) disease-free survival, and (D) local recurrence rate between laparoscopic and robotic TME (all *P* values were not significant). TME = total mesorectal excision.

There was no significant difference in the overall tumor recurrence between the groups (*P* = 0.660). Tumor recurrence, including both local and systemic recurrences, occurred in 53 patients (19.1%) in the L-TME group and in 49 patients (17.3%) in the R-TME group during the study period. Among all patients who underwent L-TME, 6 patients (2.2%) had local recurrence without systemic recurrence, and 44 patients (15.8%) had systemic recurrence without local recurrence. In the R-TME group, 5 patients (1.8%) had local recurrence without systemic recurrence and 34 patients (12.2%) had systemic recurrence. Local and systemic recurrence was observed in 3 patients (1.1%) in the L-TME group and 9 patients (3.3%) in the R-TME group. The overall 5-year LRR (Figure 3) and SRR were 3.9% and 18.0% in the L-TME group and 5.9% and 16.3% in R-TME group, respectively. In the multivariate analysis of risk factors for local recurrence, postoperative complications higher than grade III (*P* = 0.005; HR = 3.674; 95% CI 1.485–9.090) and CRM involvement (*P* = 0.002; HR = 5.653; 95% CI 1.877–17.028) were found to be independent prognostic factors, whereas tumor size >3.0 cm (*P* = 0.049; HR = 1.600; 95% CI 1.001–2.556), stage III disease (*P* < 0.001; HR = 3.232; 95% CI 1.964–5.318), and CRM involvement (*P* = 0.034; HR = 2.090; 95% CI 1.059–4.127) were found to be independent prognostic factors for systemic recurrence (Table 5).

**TABLE 5:**
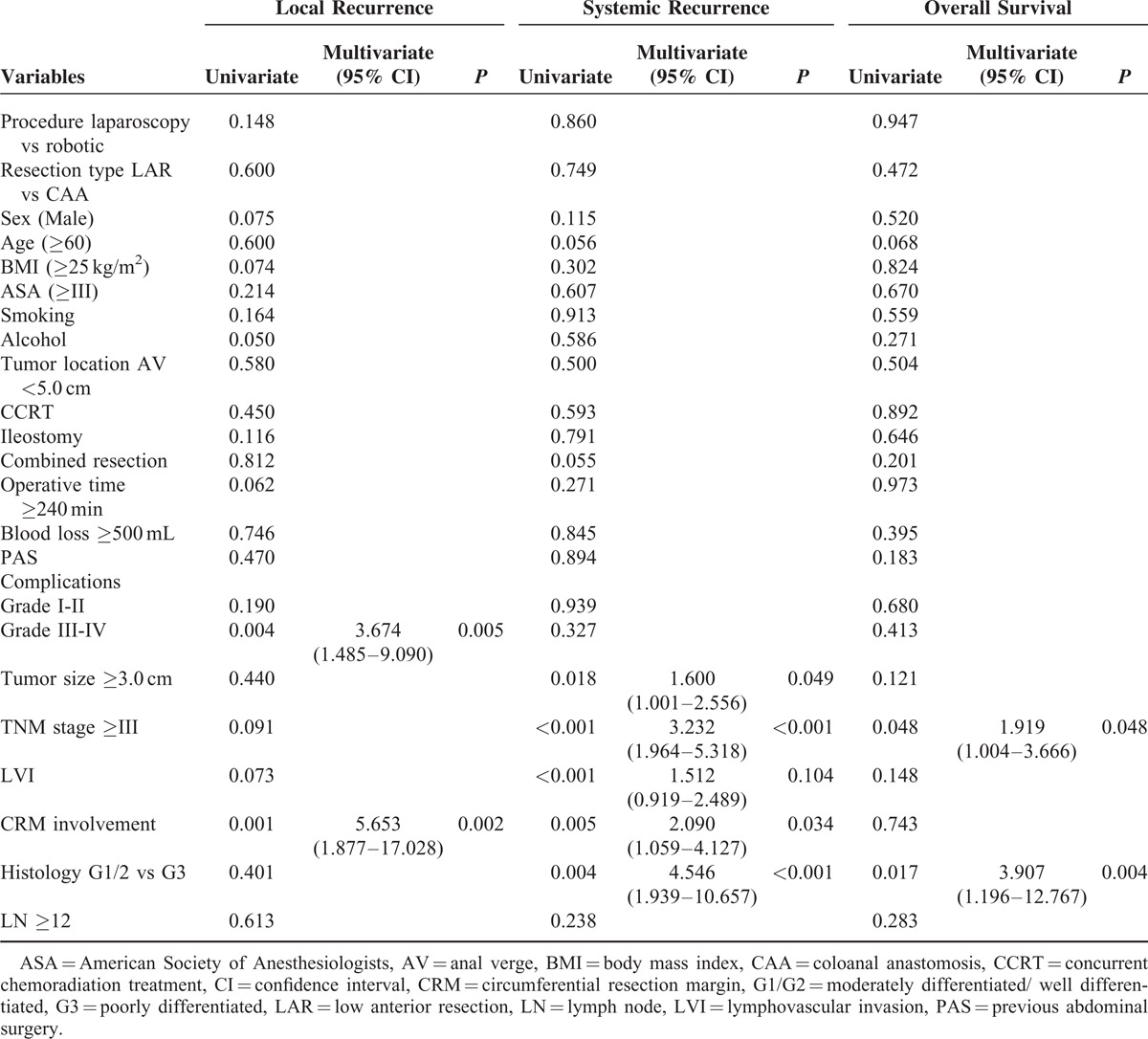
Univariate and Multivariate Analysis of Affecting Factors for Tumor Recurrence and Overall Survival

In terms of the long-term oncologic outcomes, 5-year OS (L-TME vs R-TME: 93.1% vs 92.2%; *P* = 0.422), 5-year DSS (L-TME vs R-TME: 95.5% vs 93.6%; *P* = 0.120) and 5-year DFS (L-TME vs R-TME: 79.6% vs 81.8%; *P* = 0.538) were found to be similar between the groups (Figure 3). Additionally, when the patients were analyzed according to the disease stage, there were no significant differences between the groups. The 5-year OS rates for L-TME versus R-TME for stage 0, I, II, and III disease were 100.0% vs 90.9% (*P* = 0.062), 98.7% versus 95.4% (*P* = 0.345), 89.0% versus 91.6% (*P* = 0.409), and 89.0% versus 91.2% (*P* = 0.577), respectively. The 5-year DSS rates for L-TME versus R-TME for stage 0, I, II, and III disease were 100.0% versus 93.0% (*P* = 0.104), 100.0% versus 97.9% (*P* = 0.350), 95.8% versus 92.9% (*P* = 0.594), and 90.0% versu1s 91.2% (*P* = 0.417), respectively. The 5-year DFS rates for L-TME versus R-TME for stage 0, I, II, and III disease were 90.5% versus 95.4% (*P* = 0.504), 88.3% versus 89.5% (*P* = 0.947), 86.2% versus 81.2% (*P* = 0.437), and 63.2% versus 68.2% (*P* = 0.410), respectively. In the multivariate analysis, prognostic factors impacting the 5-year OS were stage III disease (*P* = 0.048; HR = 1.919; 95% CI 1.004–3.666) and high grade of differentiation (*P* = 0.004; HR = 3.907; 95% CI 1.196–12.767) (Table 5).

## DISCUSSION

The aim of this study was to compare the short- and long-term outcomes in an attempt to investigate the impact of R-TME on rectal cancer. This case-matched study, which included >500 patients with long-term follow-up data, revealed no significant differences in the short-term outcomes, rate of postoperative complications, oncologic quality, tumor recurrence, or the 5-year OS, and the DFS rates. However, our study showed that R-TME was significantly associated with a much lower incidence of late voiding dysfunction than L-TME (0.7% vs 4.3%, *P* = 0.012). Although the introduction of TME has resulted in improved genitourinary functional preservation, most colorectal surgeons are still faced with challenging conditions such as injuries to the hypogastric nerves and/or the sacral splanchnic nerve during pelvic dissection.^21,22^ Recently, Kim et al^23^ showed that R-TME was significantly associated with earlier recovery in voiding and sexual function compared with L-TME. Similarly, Luca F et al^24^ demonstrated that R-TME allowed for better preservation of urinary and sexual functions compared with L-TME, and concluded that this may be attributed to superior movement of the wristed instruments, and to the precise pelvic dissection with better dexterity under stable magnified view. Theoretically, the use of a robotic system can decrease the risk of collateral injury to the pelvic autonomic nerves. However, there are currently only limited studies evaluating the impact of robotic technology on urogenital complications after TME. Thus, whether these theoretical advantages of R-TME translate into significant favorable urogenital function still remains to be determined. Randomized clinical trials such as the COLRAR trial (NCT01423214) and ROLARR trial (NCT01196000) are currently ongoing to clarify this issue, and more objective data may be obtained from these clinical trials in the future.

Owing to the many advantages of MIS, surgeons previously performing rectal surgery using the open technique have begun to show interest in the feasibility of transitioning into the MIS technique. On the basis of this trend, many colorectal surgeons have evaluated the true benefits of robotic technology, which overcomes the technical limitations of L-TME. Kang et al^25^ showed that R-TME in the treatment of mid or low rectal cancer was associated with decreased analgesia use, less postoperative pain, and a shorter hospital stay. Recently, Park et al furthermore reported that the rate of conversion was significantly lower for R-TME than L-TME (0.0% vs 7.1%, *P* = 0.003).^16^ Similarly, the short-term outcomes from 2 meta-analyses revealed that R-TME was associated with a significantly lower conversion rate and equivalent oncologic adequacy compared with L-TME.^26,27^ However, there were several possible confounding factors, especially regarding the conversion rate. Moreover, Kang et al showed similar conversion rates between R-TME and L-TME (0.6% vs 1.8%, *P* *=* 0.623), and similarly, the rates of conversion in this study did not significantly differ between the groups (R-TME vs L-TME: 0.4% vs 0.7%, *P* = 1.000). This result may be explained by several factors: first, the designs of most of the previous studies were heterogeneous. In the present study, propensity score matching with 8 clinical factors that may potentially affect the surgical outcomes was used to reduce patient selection biases. Although there are inevitable hidden selection biases from unmatched variables, propensity score matching is a useful method for reducing selection bias between groups, and we believe that our case-matched study design may have potentially contributed to the similar conversion rates among the groups. Second, in most of the previous reports, the proficiency of TME by a single or multiple surgeons was not assessed using objective parameters. Although CUSUM score graphs was not provided in the text, we analyzed a learning curve of R-TME to minimize confounding surgeon-related factors in this study. Our results of the CUSUM analysis indicated that no definite peak point of the learning curve for R-TME was found in 2 skilled surgeons with previous extensive laparoscopic experience, and this may, at least partly, explain the equivalent conversion rate between the groups. However, although CUSUM plots are useful for evaluating the degree of a surgeon's proficiency, the current study did not offer a full explanation for the low rate of conversion in the R-TME group. Therefore, further randomized clinical trials are required to elucidate the technical benefits of robotic surgery by using conversion rate as an endpoint.

Moreover, questions still remain whether R-TME have any influence on the oncologic outcomes and postoperative morbidity in rectal cancer patients, as compared with L-TME. Although the currently available data are limited, recent studies have reported at least equivalency between L-TME and R-TME in terms of critical perioperative outcomes such as postoperative complications, CRM involvement rate, and lymph node yield.^13,16,25,28^ Consistent with these previous studies, our study showed equivalent outcomes between L-TME and R-TME, including regarding the quality of oncologic resection, such as CRM involvement (L-TME vs R-TME: 4.7% vs 5.0%, *P* = 1.000), lymph node harvest (L-TME vs R-TME: 16.2 ± 8.1 vs 15.0 ± 8.1, *P* = 0.069), and rate of postoperative complications. CRM, which has been reported to be associated with local recurrence, can be used as an indicator of the quality of TME.^29,30^ Accordingly, in this study, CRM involvement was found to be significantly associated with tumor recurrence in both techniques.

In terms of the long-term oncologic aspects, previous comparative studies have reported similar 3-year oncologic outcomes between R-TME and L-TME.^28,31,32^ Recently, Baik et al reported that there was no significant difference in terms of the 5-year LRR and OS and DFS rates (2.3% vs 1.2%, *P* = 0.649; 92.8% v 93.5%, *P* = 0.829; 81.9% vs 78.7%, *P* = 0.547, respectively) between hybrid R-TME and L-TME.^16^ Similarly, the present study also demonstrated similar long-term oncologic outcomes between R-TME and L-TME in terms of the 5-year LRR (5.9% vs 3.9%, *P* = 0.313) and OS (92.2% vs 93.1%, *P* = 0.422) and DFS (81.8% vs 79.6%, *P* = 0538) rates. Based on the data presented in our study, we conclude that both R-TME and L-TME provided acceptable long-term oncologic outcomes in rectal surgery. However, similar to in the previous reports, R-TME did not show any superior oncologic benefits over L-TME in this study.

In the present study, major complications occurred in 68 (12.2%) patients. Among these patients, 59 (86.7%) patients who underwent radiologic intervention or surgical treatment for anastomotic leakage were included in Grade III–IV. A few previous studies have demonstrated that major complications such as anastomotic leakage influence both local recurrence and decreased survival rate.^33–35^ Consistent with these previous results, the results of the present study showed that the presence of major complications (grade III–IV) (*P* = 0.005; HR = 3.674) was an independent prognostic factor for local recurrence in the multivariate analysis. The reason for the association between anastomotic leakage and local recurrence is still unclear. However, several potential mechanisms responsible for local recurrence, such as deposition of viable tumor cells into the pelvis or shedding into the bowel lumen, have been previously reported.^36,37^ In addition, major complications may potentially contribute to delayed adjuvant chemotherapy, further affecting the patient survival. Based on these facts, the technical feasibility and oncologic safety should be considered when deciding on adopting robotic technology.

This case-matched study aimed to elucidate the potential advantages of robotic surgery in patients with rectal cancer by comparing totally robotic and L-TME. However, the results of R-TME in the current study did not show any superior long-term oncologic outcomes compared with L-TME. In terms of overcoming the technical difficulty associated with narrow pelvis and in terms of the potential functional benefits such as earlier recovery of voiding function, the robotic technique is theoretically an attractive treatment option to both surgeons and patients. However, based on our findings herein, major drawbacks of R-TME include a significant longer operation time as well as the lack of substantial superiority over L-TME. In addition, although our study did not assess the cost efficiency of robotic surgery, the higher cost of robotics versus laparoscopy should be considered when adopting robotic surgery in patients with rectal cancer.

In conclusion, both L-TME and R-TME are feasible approaches for the treatment of rectal cancer in terms of the postoperative complications, and short- and long-term outcomes. However, any true benefits of robotic surgery are still questionable, and whether R-TME can be justified as a standard procedure in rectal cancer patients needs to be further clarified.

## References

[1] Clinical Outcomes of Surgical Therapy Study G. A comparison of laparoscopically assisted and open colectomy for colon cancer. *N Engl J Med* 2004; 350:2050–2059. 15141043 10.1056/NEJMoa032651

[2] Guillou PJ Quirke P Thorpe H Short-term endpoints of conventional versus laparoscopic-assisted surgery in patients with colorectal cancer (MRC CLASICC trial): multicentre, randomised controlled trial. *Lancet* 2005; 365:1718–1726. 15894098 10.1016/S0140-6736(05)66545-2

[3] Jayne DG Guillou PJ Thorpe H Randomized trial of laparoscopic-assisted resection of colorectal carcinoma: 3-year results of the UK MRC CLASICC Trial Group. *J Clin Oncol* 2007; 25:3061–3068. 17634484 10.1200/JCO.2006.09.7758

[4] Law WL Lee YM Choi HK Impact of laparoscopic resection for colorectal cancer on operative outcomes and survival. *Ann Surg* 2007; 245:1–7. 17197957 10.1097/01.sla.0000218170.41992.23 PMC1867940

[5] Leung KL Kwok SP Lam SC Laparoscopic resection of rectosigmoid carcinoma: prospective randomised trial. *Lancet* 2004; 363:1187–1192. 15081650 10.1016/S0140-6736(04)15947-3

[6] Kang SB Park JW Jeong SY Open versus laparoscopic surgery for mid or low rectal cancer after neoadjuvant chemoradiotherapy (COREAN trial): short-term outcomes of an open-label randomised controlled trial. *Lancet Oncol* 2010; 11:637–645. 20610322 10.1016/S1470-2045(10)70131-5

[7] van der Pas MH Haglind E Cuesta MA Laparoscopic versus open surgery for rectal cancer (COLOR II): short-term outcomes of a randomised, phase 3 trial. *Lancet Oncol* 2013; 14:210–218. 23395398 10.1016/S1470-2045(13)70016-0

[8] Green BL Marshall HC Collinson F Long-term follow-up of the Medical Research Council CLASICC trial of conventional versus laparoscopically assisted resection in colorectal cancer. *Br J Surg* 2013; 100:75–82. 23132548 10.1002/bjs.8945

[9] Huang MJ Liang JL Wang H Laparoscopic-assisted versus open surgery for rectal cancer: a meta-analysis of randomized controlled trials on oncologic adequacy of resection and long-term oncologic outcomes. *Int J Colorectal Dis* 2011; 26:415–421. 21174107 10.1007/s00384-010-1091-6

[10] Laurent C Leblanc F Wutrich P Laparoscopic versus open surgery for rectal cancer: long-term oncologic results. *Ann Surg* 2009; 250:54–61. 19561481 10.1097/SLA.0b013e3181ad6511

[11] Ng SS Lee JF Yiu RY Long-term oncologic outcomes of laparoscopic versus open surgery for rectal cancer: a pooled analysis of 3 randomized controlled trials. *Ann Surg* 2014; 259:139–147. 23598381 10.1097/SLA.0b013e31828fe119

[12] Bretagnol F Lelong B Laurent C The oncological safety of laparoscopic total mesorectal excision with sphincter preservation for rectal carcinoma. *Surg Endosc* 2005; 19:892–896. 15920688 10.1007/s00464-004-2228-x

[13] Baik SH Kwon HY Kim JS Robotic versus laparoscopic low anterior resection of rectal cancer: short-term outcome of a prospective comparative study. *Ann Surg Oncol* 2009; 16:1480–1487. 19290486 10.1245/s10434-009-0435-3

[14] D’Annibale A Morpurgo E Fiscon V Robotic and laparoscopic surgery for treatment of colorectal diseases. *Dis Colon Rectum* 2004; 47:2162–2168. 15657669 10.1007/s10350-004-0711-z

[15] Baik SH Kim NK Lim DR Oncologic outcomes and perioperative clinicopathologic results after robot-assisted tumor-specific mesorectal excision for rectal cancer. *Ann Surg Oncol* 2013; 20:2625–2632. 23417433 10.1245/s10434-013-2895-8

[16] Park EJ Cho MS Baek SJ Long-term oncologic outcomes of robotic low anterior resection for rectal cancer: a comparative study with laparoscopic surgery. *Ann Surg* 2015; 261:129–137. 24662411 10.1097/SLA.0000000000000613

[17] Bokhari MB Patel CB Ramos-Valadez DI Learning curve for robotic-assisted laparoscopic colorectal surgery. *Surg Endosc* 2011; 25:855–860. 20734081 10.1007/s00464-010-1281-x PMC3044842

[18] Kim JS Cho SY Min BS Risk factors for anastomotic leakage after laparoscopic intracorporeal colorectal anastomosis with a double stapling technique. *J Am Coll Surg* 2009; 209:694–701. 19959036 10.1016/j.jamcollsurg.2009.09.021

[19] Park YA Kim JM Kim SA Totally robotic surgery for rectal cancer: from splenic flexure to pelvic floor in one setup. *Surg Endosc* 2010; 24:715–720. 19688388 10.1007/s00464-009-0656-3

[20] Dindo D Demartines N Clavien PA Classification of surgical complications: a new proposal with evaluation in a cohort of 6336 patients and results of a survey. *Ann Surg* 2004; 240:205–213. 15273542 10.1097/01.sla.0000133083.54934.ae PMC1360123

[21] Heald RJ Husband EM Ryall RD The mesorectum in rectal cancer surgery-the clue to pelvic recurrence? *Br J Surg* 1982; 69:613–616. 6751457 10.1002/bjs.1800691019

[22] Masui H Ike H Yamaguchi S Male sexual function after autonomic nerve-preserving operation for rectal cancer. *Dis Colon Rectum* 1996; 39:1140–1145. 8831531 10.1007/BF02081416

[23] Kim JY Kim NK Lee KY A comparative study of voiding and sexual function after total mesorectal excision with autonomic nerve preservation for rectal cancer: laparoscopic versus robotic surgery. *Ann Surg Oncol* 2012; 19:2485–2493. 22434245 10.1245/s10434-012-2262-1

[24] Luca F Valvo M Ghezzi TL Impact of robotic surgery on sexual and urinary functions after fully robotic nerve-sparing total mesorectal excision for rectal cancer. *Ann Surg* 2013; 257:672–678. 23001075 10.1097/SLA.0b013e318269d03b

[25] Kang J Yoon KJ Min BS The impact of robotic surgery for mid and low rectal cancer: a case-matched analysis of a 3-arm comparison-open, laparoscopic, and robotic surgery. *Ann Surg* 2013; 257:95–101. 23059496 10.1097/SLA.0b013e3182686bbd

[26] Memon S Heriot AG Murphy DG Robotic versus laparoscopic proctectomy for rectal cancer: a meta-analysis. *Ann Surg Oncol* 2012; 19:2095–2101. 22350601 10.1245/s10434-012-2270-1

[27] Trastulli S Farinella E Cirocchi R Robotic resection compared with laparoscopic rectal resection for cancer: systematic review and meta-analysis of short-term outcome. *Colorectal Dis* 2012; 14:e134–e156. 22151033 10.1111/j.1463-1318.2011.02907.x

[28] Baek SJ Al-Asari S Jeong DH Robotic versus laparoscopic coloanal anastomosis with or without intersphincteric resection for rectal cancer. *Surg Endosc* 2013; 27:4157–4163. 23708725 10.1007/s00464-013-3014-4

[29] Birbeck KF Macklin CP Tiffin NJ Rates of circumferential resection margin involvement vary between surgeons and predict outcomes in rectal cancer surgery. *Ann Surg* 2002; 235:449–457. 11923599 10.1097/00000658-200204000-00001 PMC1422458

[30] Nagtegaal ID Quirke P What is the role for the circumferential margin in the modern treatment of rectal cancer? *J Clin Oncol* 2008; 26:303–312. 18182672 10.1200/JCO.2007.12.7027

[31] Baek JH McKenzie S Garcia-Aguilar J Oncologic outcomes of robotic-assisted total mesorectal excision for the treatment of rectal cancer. *Ann Surg* 2010; 251:882–886. 20395863 10.1097/SLA.0b013e3181c79114

[32] Pigazzi A Luca F Patriti A Multicentric study on robotic tumor-specific mesorectal excision for the treatment of rectal cancer. *Ann Surg Oncol* 2010; 17:1614–1620. 20087780 10.1245/s10434-010-0909-3

[33] Branagan G Finnis D Wessex Colorectal Cancer Audit Working G. Prognosis after anastomotic leakage in colorectal surgery. *Dis Colon Rectum* 2005; 48:1021–1026. 15789125 10.1007/s10350-004-0869-4

[34] Merkel S Wang WY Schmidt O Locoregional recurrence in patients with anastomotic leakage after anterior resection for rectal carcinoma. *Colorectal Dis* 2001; 3:154–160. 12790981 10.1046/j.1463-1318.2001.00232.x

[35] Walker KG Bell SW Rickard MJ Anastomotic leakage is predictive of diminished survival after potentially curative resection for colorectal cancer. *Ann Surg* 2004; 240:255–259. 15273549 10.1097/01.sla.0000133186.81222.08 PMC1356401

[36] Balkwill F Mantovani A Inflammation and cancer: back to Virchow? *Lancet* 2001; 357:539–545. 11229684 10.1016/S0140-6736(00)04046-0

[37] Bell SW Walker KG Rickard MJ Anastomotic leakage after curative anterior resection results in a higher prevalence of local recurrence. *Br J Surg* 2003; 90:1261–1266. 14515297 10.1002/bjs.4219

